# A 4-Week Mobile App–Based Telerehabilitation Program vs Conventional In-Person Rehabilitation in Older Adults With Sarcopenia: Randomized Controlled Trial

**DOI:** 10.2196/67846

**Published:** 2025-01-24

**Authors:** Lu Zhang, Ying Ge, Wowa Zhao, Xuan Shu, Lin Kang, Qiumei Wang, Ying Liu

**Affiliations:** 1 Department of Rehabilitation Medicine Peking Union Medical College Hospital Chinese Academy of Medical Sciences and Peking Union Medical College Beijing China; 2 Department of Geriatric Medicine Peking Union Medical College Hospital Chinese Academy of Medical Sciences and Peking Union Medical College Beijing China

**Keywords:** telerehabilitation, elderly, sarcopenia, resistance exercise, rehabilitation, gerontology, aging, randomized controlled trial, rehabilitation training, body composition, strength, balance, cardiorespiratory endurance, self-care, physical therapy

## Abstract

**Background:**

Sarcopenia is closely associated with a poor quality of life and mortality, and its prevention and treatment represent a critical area of research. Resistance training is an effective treatment for older adults with sarcopenia. However, they often face challenges when receiving traditional rehabilitation treatments at hospitals.

**Objective:**

We aimed to compare the effects of a digital rehabilitation program with those of traditional therapist-supervised rehabilitation training on older adults with sarcopenia.

**Methods:**

In total, 58 older adults with sarcopenia were recruited offline and randomized (1:1) into 2 groups: the telerehabilitation group (TRG, n=29, 50%) and the in-person rehabilitation group (IRG, n=29, 50%). Both groups underwent 4-week resistance training targeting 6 major muscle groups. The TRG received exercise guidance via a mobile app, while the IRG received in-person training from a therapist. Offline assessments of body composition, grip strength, and balance using the 30-Second Arm Curl Test (30SACT), 30-Second Sitting-to-Rising Test (30SSRT), quadriceps femoris extension peak torque (EPT) and extension total power (ETP), Berg Balance Scale (BBS), Timed Up-and-Go Test (TUGT), 6-Minute Walk Test (6MWT), and Instrumental Activities of Daily Living (IADL) scale, were conducted before and after the intervention.

**Results:**

Of the 58 patients, 51 (88%; TRG: n=24, 47%; IRG: n=27, 53%) completed the trial. After 4 weeks of intervention, the mean grip strength increased from 18.10 (SD 5.56) to 19.92 (SD 5.90) kg in the TRG (*P*=.02) and from 18.59 (SD 5.95) to 19.59 (SD 6.11) kg in the IRG (*P*=.01). The 30SACT and 30SSRT scores increased from 12.48 (SD 2.68) to 14.94 (SD 3.68) times (*P*=.01) and from 15.16 (SD 7.23) to 16.58 (SD 8.42) times (*P*=.045), respectively, in the TRG and from 12.25 (SD 4.19) to 14.68 (SD 4.36) times (*P*=.003) and from 14.31 (SD 4.04) to 16.25 (SD 4.91) times (*P*=.01), respectively, in the IRG. The quadriceps femoris EPT increased from 26.19 (SD 10.26) to 35.00 (SD 13.74) Nm (*P*=.004) in the TRG and from 26.95 (SD 11.81) to 32.74 (SD 12.33) Nm (*P*=.003) in the IRG. The BBS scores significantly improved in both groups (*P*<.001), with the mean TRG score increasing by 3.19 (SD 2.86) points and the mean IRG score by 3.06 (SD 2.44) points. Neither group exhibited significant within-group changes on the TUGT or the 6MWT. Both groups reported significant improvements in the IADL (TRG: *P*=.04; IRG: *P*=.02). Between-group comparisons revealed no significant differences in changes in all indicators.

**Conclusions:**

A 4-week remote resistance training program is effective in improving strength, balance, and the IADL in older adults with sarcopenia, with effects comparable to rehabilitation supervised by a physical therapist. Telerehabilitation may be a convenient and effective alternative for older adults with sarcopenia who have limited access to rehabilitation resources.

**Trial Registration:**

ChiCTR 2300071648; https://www.chictr.org.cn/showprojEN.html?proj=196313

## Introduction

Muscle reduction typically progresses gradually between the ages of 40 and 50 years but accelerates rapidly after the age of 60 years, with approximately 30% of skeletal muscle mass lost between the ages of 50 and 80 years [1,2]. Sarcopenia is characterized by a substantial reduction in skeletal muscle mass and function. This condition heightens the risk of falls, fractures, and physical disability and is closely linked to a poorer quality of life, as well as increased mortality rates [3,4].

According to a systematic review from 2022, the prevalence of sarcopenia worldwide is estimated to be 8%-36% in individuals under the age of 60 years and 10%-27% in those over 60 years [5]. Based on the literature from 2016 to 2019, in Asian countries, the prevalence ranges from 5.5% to 25.7% [6]. More specifically, in China, recent data suggest that the overall prevalence of sarcopenia among individuals aged 60 years and older is about 20.7%, and this rate has been rising over the past 5 years [7]. Thus, the prevention and treatment of sarcopenia represent a critical area of research, with significant implications for the health of the older population.

Resistance training has been shown to enhance muscle strength, aerobic endurance, and muscle mass [8]. This includes various exercises using free weights, machines, resistance bands, or body weight. Resistance training has demonstrated notable efficacy in improving strength in patients with sarcopenia [9-12]. Guidelines from the World Health Organization, as well as those from the United Kingdom and Australia, recommend 2-3 sessions of resistance training per week to prevent and manage sarcopenia [13,14]. Furthermore, consistent adherence to exercise is crucial, as irregular or intermittent training may impair treatment outcomes [15].

However, older adults (defined in this study as individuals aged 60 years or older, according to standards set by the World Health Organization [16]) with sarcopenia often face challenges when engaging in resistance training. In middle-income countries in particular, access to professional rehabilitation services is limited due to inadequate rehabilitation facilities, transportation barriers, financial constraints, and diminished physical ability, all of which reduce patient participation [14,17]. Additionally, older adults tend to have low adherence to self-guided home training programs, largely due to the lack of professional supervision, fear of injury, and low motivation [9].

Given these challenges, digital programs delivered through mobile apps offer a potential solution, allowing patients to receive professional guidance and monitoring from the comfort of their homes. This approach could alleviate the burden on hospital resources and improve patient adherence to training programs [18]. Recent studies have shown that home-based programs can have a positive impact on improving the function and quality of life of older adults [19,20]. Therefore, this study aimed to compare the effects of a digital rehabilitation program with traditional therapist-supervised rehabilitation training in older adults with sarcopenia, addressing the aforementioned challenges.

## Methods

### Study Design

This single-center randomized controlled trial (RCT; Multimedia Appendix 1) was conducted at the Peking Union Medical College Hospital, affiliated with the Chinese Academy of Medical Sciences. The study is registered with the Chinese Clinical Trial Registry (registration number: ChiCTR 2300071648).

### Ethical Considerations

This study was approved by the Ethics Committee of the Peking Union Medical College Hospital (approval number: JS-2648). All participants provided written informed consent prior to enrollment, ensuring their voluntary participation with a full understanding of the study’s objectives, procedures, and potential risks. All personal data collected were anonymized and stored in encrypted electronic databases accessible only to the research team members. Neither the manuscript nor the supplementary materials include any identifiable information or photographs of individual participants. Additionally, no form of financial compensation was provided to participants; participation was entirely voluntary and would not influence their routine medical services.

### Participants

All participants in this study were recruited offline from the Peking Union Medical College Hospital, a large comprehensive hospital. The specific process was as follows: First, the researchers posted recruitment advertisements in the hospital’s outpatient hall. Second, potential participants contacted the recruitment team (2 geriatricians and a rehabilitation physician with extensive experience in sarcopenia) over the phone, and the recruitment team conducted an initial telephone screening. Third, potential participants who passed the initial telephone screening were invited to the hospital for a face-to-face detailed assessment, including medical history taking, physical examination, and necessary laboratory tests. Finally, if the conditions of the potential participants met all the eligibility criteria, they were formally included in the study after obtaining written informed consent. During the informed consent process, participants were not informed whether they were in the intervention group or the control group in order to avoid bias. The inclusion and exclusion criteria are outlined in Textbox 1.

**Inclusion and exclusion criteria for the study.**

**Inclusion Criteria**

- Diagnosed with sarcopenia based on the 2019 Asian Working Group for Sarcopenia (AWGS) criteria [6]
- Age between 60 and 80 years
- Able to operate a smartphone and follow the exercise regimen, with sufficient literacy in the local language to navigate a Chinese language mobile app
- No participation in any rehabilitation program within the past 30 days

**Exclusion Criteria**

- Severe cognitive impairment (Mini-Mental State Examination [MMSE] score<24), hearing impairment, or vision impairment
- Severe neurological, musculoskeletal, or organ (cardiovascular, pulmonary, hepatic, renal) diseases or malignant tumors
- Uncontrolled hypertension, unstable metabolic diseases, or any other condition deemed inappropriate for participation by the researchers

### Sample Size Calculation

The sample size was calculated using PASS 15 software (NCSS, LLC). Based on the principle of noninferiority RCTs and previous clinical studies [21], the mean difference in the grip strength between the home-based exercise group and the therapist supervision–based exercise group was 3, the SD was estimated to be 4 for both groups, and the noninferiority margin for the grip was 1. A sample size of 46 was required based on a 1-sided α=.025 and β=.1, and a sample size of 58 was required to account for a 20% dropout rate. The formula used is the following:



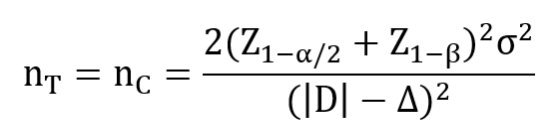



### Randomization

A total of 58 participants were randomly assigned to either the telerehabilitation group (TRG, n=29, 50%) or the in-person rehabilitation group (IRG, n=29, 50%) using a randomization platform. Based on the platform’s results (eg, C, T, C, T, T, C), slips of paper labeled “T” and “C” were placed into sealed, opaque, and uniformly sized envelopes. After completing baseline measurements, the envelopes were opened in sequence to reveal group assignments. The allocation sequence was generated using a blocked randomization model by 2 researchers who were not involved in the study.

### Exercise Program

**Figure 1:**
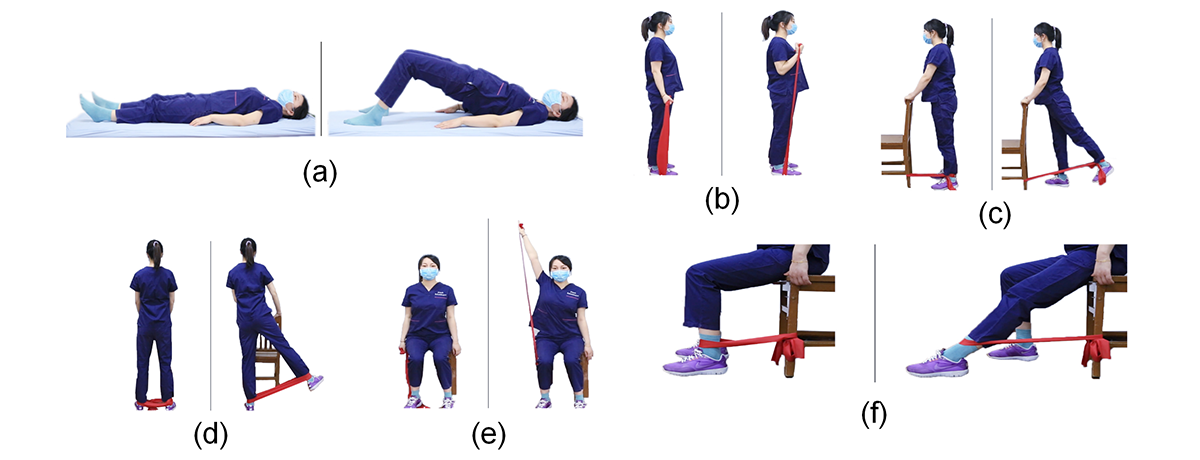
Exercises included (a) glute bridge, (b) resisted elbow flexion, (c) resisted hip extension, (d) resisted hip abduction, (e) resisted shoulder abduction, and (f) resisted knee extension.

The training program for patients with sarcopenia in both the TRG and the IRG was identical. The program consisted of 6 resistance exercises targeting specific muscle groups, including back extensors, biceps brachii, gluteus maximus, gluteus medius, the deltoid, and quadriceps femoris. The specific exercises included glute bridges, resisted elbow flexion, hip extension, hip abduction, shoulder abduction, and knee extension (Figure 1). Participants performed resistance training 3 times a week, with each exercise performed in 3 sets of 10 repetitions per set, over a 4-week period (Multimedia Appendix 2). Effective training intensity was ensured through the use of the Borg rating of perceived exertion (RPE), with a target RPE of 12-14 at the end of each exercise session. Each session included approximately 10 minutes of warm-up, 40 minutes of resistance training, and 10 minutes of stretching, totaling around 1 hour.

#### Intervention Group (TRG)

**Figure 2:**
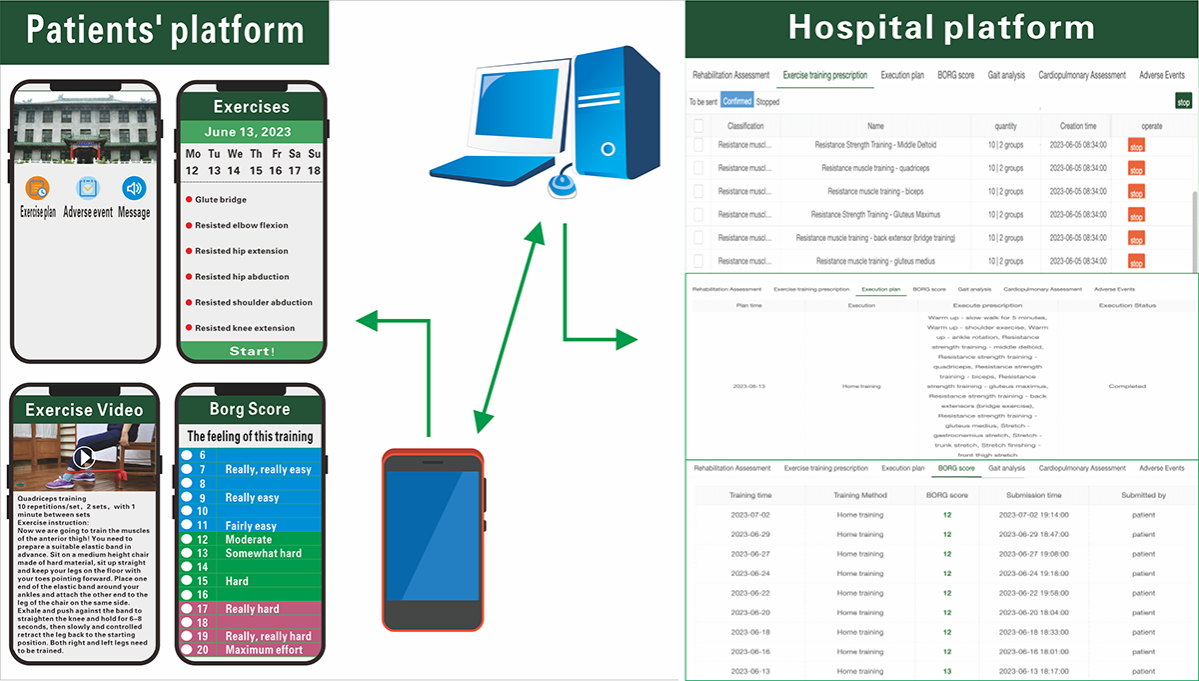
The 3 different parts of the telerehabilitation system (the language of the interface is translated from Chinese). The doctor’s portal can be used to create and modify exercises, monitor training progress, and view patient data. Patients can use the user portal to complete the prescribed exercises and provide feedback to physical therapists. Finally, the transmitter portal encrypts and transmits the data collected, ensuring the overall system’s integrity.

Before initiating at-home training, each participant attended an individual, face-to-face introductory session at the hospital’s rehabilitation outpatient clinic. A physical therapist demonstrated the exercises, ensured the participants understood the correct technique, and assisted them with downloading and registering the mobile app on their personal smartphones. Each participant received a personalized exercise protocol via the app and was provided with resistance bands with appropriate tension levels based on their initial assessment. Following this initial session, participants performed the exercises independently at home, guided by instructional videos and written instructions embedded in the app, which were marked with the hospital’s logo (Figure 2).

The telerehabilitation system comprised 3 main components:

- Physician portal: Accessed by the medical team to upload exercise videos and protocols and track patient adherence.
- User portal (patient interface): Accessed via a password-protected smartphone app in the local (Chinese) language. Patients navigated using simple menus and icons to view exercise instructions, watch demonstration videos, and report session completion. The interface includes step-by-step written instructions and demonstration videos. Patients were required to input their RPE values after each session.
- Transmission portal: A secure data transmission platform integrated within the system automatically records the exercise duration, frequency, and patient-reported RPE. Patients were instructed to report any adverse events through the app immediately, triggering an alert to the research team.

#### Control Group (IRG)

Participants assigned to the IRG attended scheduled sessions in the hospital’s rehabilitation department, which is a typical form of training currently in practice. A physical therapist supervised each session, providing on-site guidance, feedback, and adjustments to ensure correct exercise performance. The IRG followed the same exercise protocol, frequency, and intensity guidelines as the TRG, but all training took place face to face under professional supervision.

### Measures

All assessments were conducted by a dedicated blinded physical therapist with professional certification. This individual conducted all tests within 3 days in the hospital before and after the 4-week intervention for every participant.

#### Body Composition

Body composition was measured using bioelectrical impedance analysis (BIA-101 Anniversary Sport Edition, Akern-RJL Systems), with specific metrics including the total skeletal muscle mass (TSM), body fat percentage (BFP), and skeletal muscle mass index (SMI).

#### Strength

Strength was assessed using grip strength, the 30-Second Arm Curl Test (30SACT), the 30-Second Sitting-to-Rising Test (30SSRT), and the quadriceps femoris extension peak torque (EPT) and extension total power (ETP). For the grip strength test, participants used their dominant hand to apply the maximum pressure to the dynamometer handle (Jamar Hydraulic T, Patterson Medical). In the 30SACT, participants sat with their upper arms pressed against their torso while holding 8 lb dumbbells (men) and 5 lb dumbbells (women). They were instructed to perform elbow curls as quickly as possible for 30 seconds. In the 30SSRT, participants sat on a 43-cm-high chair and alternated between sitting and standing as many times as possible within 30 seconds. Each of these tests was performed 3 times, with a 1-minute rest between trials, and the maximum value was recorded as the final result.

Quadriceps extension strength indices were measured using an isokinetic dynamometer (ISOMOVE, TecnoBody). The seat of the dynamometer was reclined at approximately 85°, with the participant’s thigh, pelvis, and torso secured by straps. The rotation axis of the device was aligned with the lateral epicondyle of the femur, and the lever pad was positioned 3 cm above the lateral malleolus. The test consisted of 5 maximal repetitions at a speed of 20°/second, during which participants extended their knees as fast as possible against the resistance shown on the screen, reaching the target end point indicated by the graphical display.

#### Balance, Cardiopulmonary Endurance, and Self-Care Ability

Balance was assessed using the Berg Balance Scale (BBS) and the Timed Up-and-Go Test (TUGT). The BBS evaluated 14 tasks, with each task scored from 0 (unable to complete) to 4 (completed independently), and the total score represented the sum of all individual task scores. The TUGT assessed functional mobility and fall risk by timing how long it took a participant to rise from a chair, walk a short distance (3 m), turn, walk back, and sit down again.

Cardiopulmonary endurance was measured using the 6-Minute Walk Test (6MWT), which quantified the distance a participant could walk on a flat, hard surface in 6 minutes. The primary outcome was the total distance walked, with longer distances indicating better cardiopulmonary function.

The Instrumental Activities of Daily Living (IADL) scale was used to assess participants’ self-care abilities.

### Statistical Analysis

Data analysis was performed using IBM SPSS version 26.0. Categorical variables were expressed as numbers, while continuous variables were presented as the mean (SD). Categorical variables were compared using the chi-square test. Paired *t* tests were used for within-group comparisons before and after the intervention, while independent-sample *t* tests were applied for comparisons between groups. *P*<.05 was considered statistically significant.

## Results

### Study Flow and General Participant Characteristics

**Figure 3:**
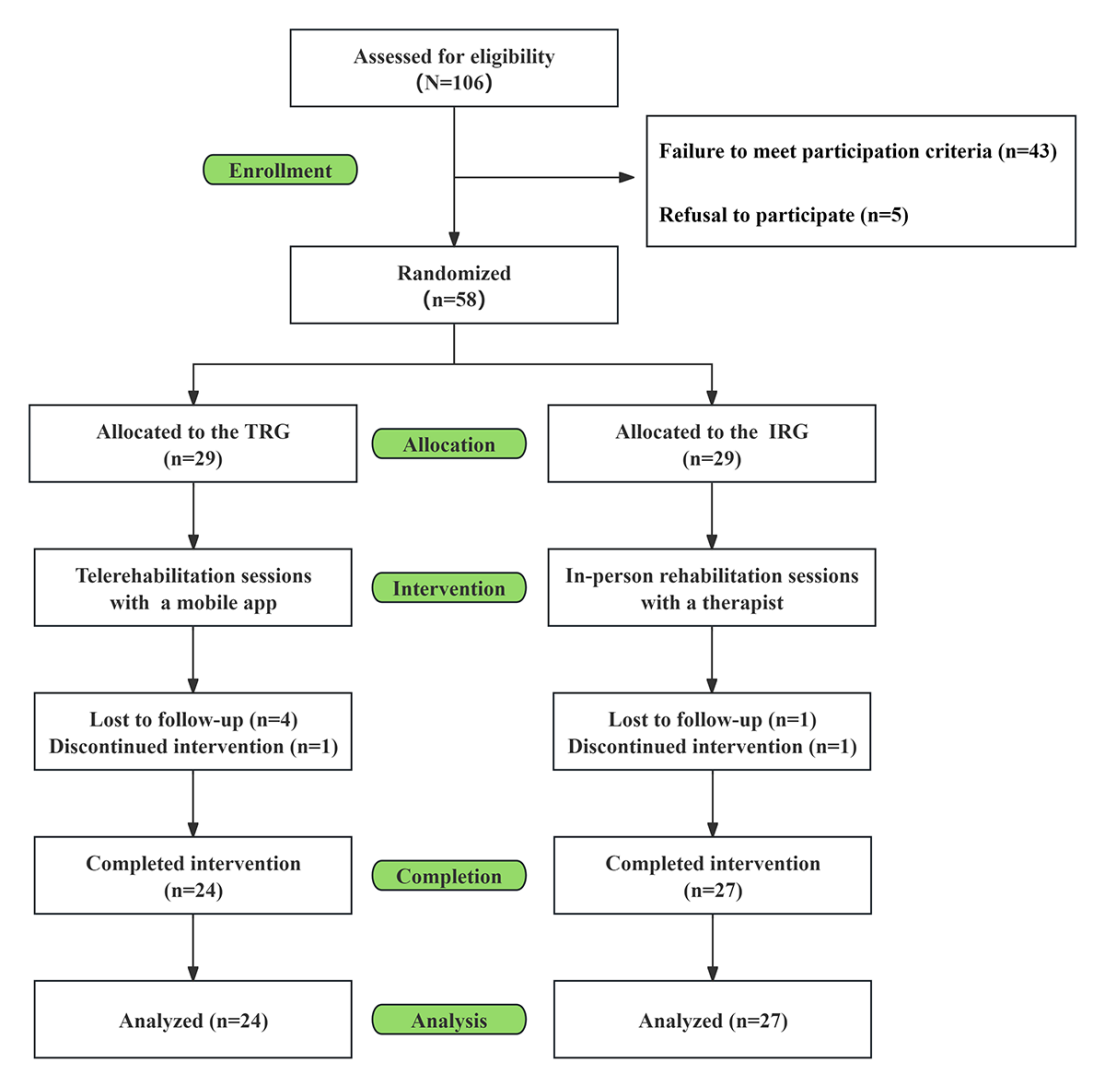
Study flow. IRG: in-person rehabilitation group; TRG: telerehabilitation group.

Between May 30, 2023, and July 1, 2024, 106 patients were assessed for eligibility. Of these, 43 (40%) patients were excluded based on the criteria, while 5 (5%) declined participation after the initial screening. As a result, 58 (55%) patients were included in the final study. As shown in Figure 3, participants were randomly assigned to 1 of 2 groups: the TRG (n=29, 50%) or the IRG (n=29, 50%).

During the study, 4 (14%) participants in the TRG and 1 (3%) participant in the IRG did not complete the 4-week follow-up. Additionally, 1 (3%) participant from each group discontinued the training due to worsening pre-existing conditions: hip pain in the TRG and elbow pain in the IRG. After discontinuing the exercise, their hip pain subsided, and the elbow pain returned to baseline levels. Ultimately, 24 (83%) patients in the TRG and 27 (93) patients in the IRG completed both the training program and the 4-week follow-up. Adherence to the exercise intervention was defined as the proportion of completed sessions relative to the total prescribed sessions, with adherence rates of 97% in the TRG and 92% in the IRG. The most commonly reported adverse effect was muscle soreness, and no exercise-related injuries or major adverse events were reported.

The baseline characteristics of the patients in the TRG and IRG were comparable. There were no significant differences between the 2 groups in terms of age, gender, height, weight, the TSM, the BFP, the SMI, grip strength, quadriceps femoris EPT, the BBS score, the 6MWT score, or the IADL score (Table 1).

**Table 1:** Participants’ (N=51) physical characteristics.

Characteristic	TRG^a^ (n=24)	IRG^b^ (n=27)	*P* value
Age (years), mean (SD)	70.47 (6.05)	69.81 (5.76)	.74
Gender (male/female), n (%)	6 (25)/18 (75)	7 (16)/20 (84)	.93
Height (cm), mean (SD)	160.73 (6.63)	161.88 (5.97)	.61
Weight (kg), mean (SD)	53.49 (7.55)	53.95 (8.14)	.87
TSM^c^ (kg/m^2^), mean (SD)	26.82 (10.04)	25.71 (8.04)	.72
BFP^d^ (%), mean (SD)	31.31 (8.34)	28.28 (6.17)	.23
SMI^e^ (kg/m^2^), mean (SD)	5.60 (0.51)	5.64 (0.77)	.88
Grip strength (kg) , mean (SD)	18.59 (5.95)	18.96 (6.57)	.87
Quadriceps femoris EPT^f^ (Nm), mean (SD)	26.19 (10.26)	26.95 (11.81)	.84
BBS^g^ score, mean (SD)	49.00 (4.18)	49.11 (4.89)	.95
6MWT^h^ score (minutes), mean (SD)	354.19 (97.56)	349.84 (97.43)	.90
IADL^i^ score, mean (SD)	21.31 (1.96)	21.42 (1.98)	.87

^a^TRG: telerehabilitation group.

^b^IRG: in-person rehabilitation group.

^c^TSM: total skeletal muscle mass.

^d^BFP: body fat percentage.

^e^SMI: skeletal muscle mass index.

^f^EPT: extension peak torque.

^g^BBS: Berg Balance Scale.

^h^6MWT: 6-Minute Walk Test.

^i^IADL: Instrumental Activities of Daily Living.

#### Body Composition

After 4 weeks, both groups showed a reduction in the BFP and an increase in the TSM and SMI, but these changes were not statistically significant (Table 2).

**Table 2:** Baseline, 4-week, and pre- and postintervention changes in the body composition.

Body composition indicators	TRG^a^ (n=24)				IRG^b^ (n=27)				Between-group difference *P* value
	Baseline, mean (SD)	Week 4, mean (SD)	Difference, mean (SD)	*P* value	Baseline, mean (SD)	Week 4, mean (SD)	Difference, mean (SD)	*P* value	
TSM^c^ (kg/m^2^)	25.71 (8.04)	27.84 (8.38)	3.04 (9.00)	.27	26.82 (10.04)	29.86 (9.83)	2.14 (8.24)	.20	.76
BFP^d^ (%)	28.28 (6.17)	27.96 (5.78)	–1.98 (6.96)	.52	31.31 (8.34)	29.33 (6.59)	–0.32 (2.09)	.27	.34
SMI^e^ (kg/m^2^)	5.64 (0.77)	5.85 (0.66)	0.30 (0.89)	.28	5.60 (0.51)	5.90 (0.99)	0.21 (0.80)	.25	.77

^a^TRG: telerehabilitation group.

^b^IRG: in-person rehabilitation group.

^c^TSM: total skeletal muscle mass.

^d^BFP: body fat percentage.

^e^SMI: skeletal muscle mass index.

#### Strength

**Figure 4:**
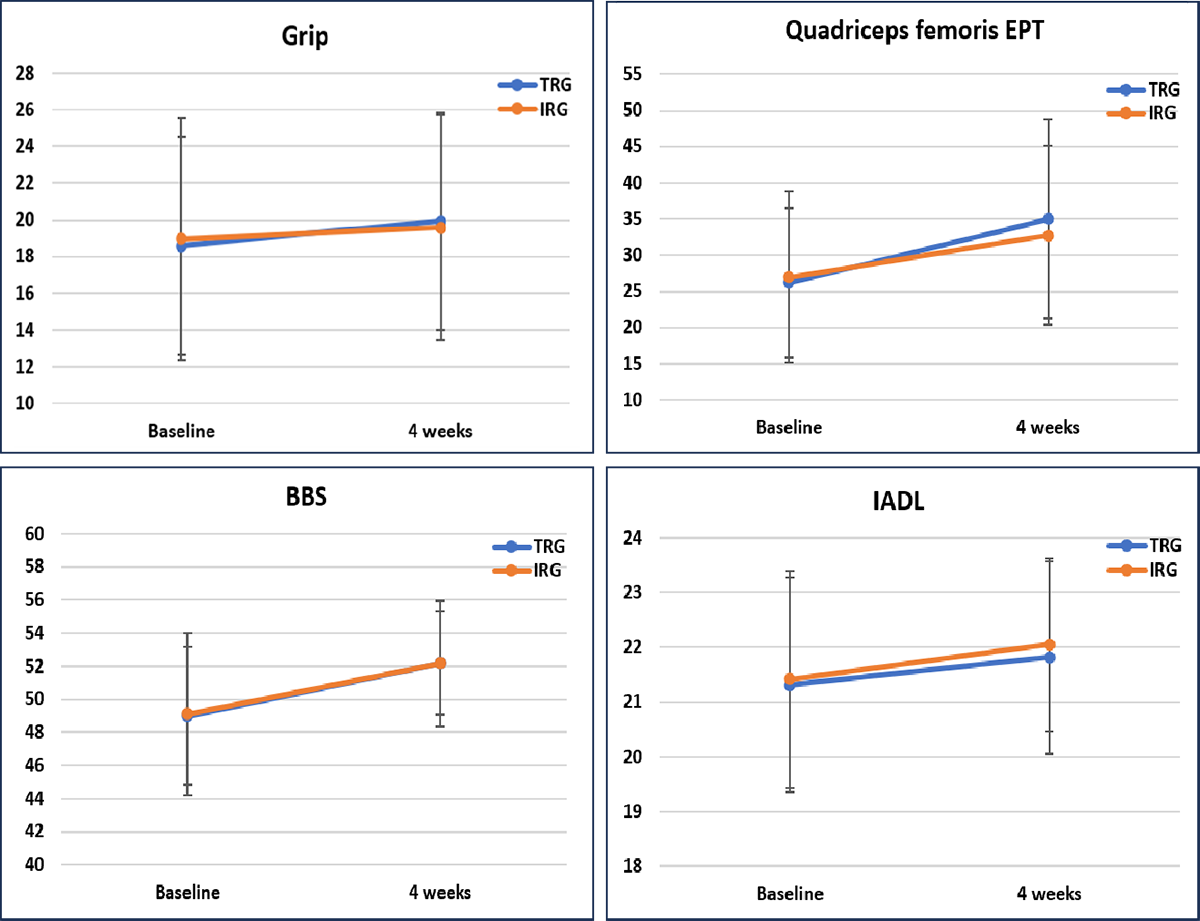
Grip strength, quadriceps femoris EPT, BBS, and IADL data at baseline and 4 weeks. Error bars represent 95% CIs. BBS: Berg Balance Scale; EPT: extension peak torque; IADL: Instrumental Activities of Daily Living; IRG: in-person rehabilitation group; TRG: telerehabilitation group.

Table 3 presents the changes in strength before and after the intervention. Both groups demonstrated significant improvements in strength (grip strength, 30SACT score, 30SSRT score, and quadriceps femoris EPT and ETP; *P*<.05), as shown in Table 3. However, there were no statistically significant differences between the groups in terms of changes in any of the strength indicators (Figure 4).

**Table 3:** Baseline, 4-week, and pre- and postintervention changes for strength.

Strength indicators	TRG^a^ (n=24)				IRG^b^ (n=27)				Between-group difference *P* value
	Baseline, mean (SD)	Week 4, mean (SD)	Difference, mean (SD)	*P* value	Baseline, mean (SD)	Week 4, mean (SD)	Difference, mean (SD)	*P* value	
Grip strength (kg)	18.10 (5.56)	19.92 (5.90)	1.33 (1.89)	.02	18.59 (5.95)	19.59 (6.11)	0.64 (2.24)	.01	.34
30SACT^c^ score (number of times)	12.48 (2.68)	14.94 (3.68)	2.13 (2.92)	.01	12.25 (4.19)	14.68 (4.36)	1.58 (1.98)	.003	.53
30SSRT^d^ score (number of times)	15.16 (7.23)	16.58 (8.42)	1.94 (2.64)	.045	14.31 (4.04)	16.25 (4.91)	1.42 (3.02)	.01	.42
Quadriceps femoris EPT^e^ (Nm)	26.19 (10.26)	35.00 (13.74)	8.81 (10.36)	.004	26.95 (11.81)	32.74 (12.33)	5.79 (7.22)	.003	.32
Quadriceps femoris ETP^f^ (J)	85.96 (26.98)	117.27 (52.48)	18.93 (40.03)	.02	97.27 (38.86)	111.89 (50.31)	19.47 (25.87)	.007	.60

^a^RG: telerehabilitation group.

^b^IRG: in-person rehabilitation group.

^c^30SACT: 30-Second Arm Curl Test.

^d^30SSRT: 30-Second Sitting-Rising Test.

^e^EPT: extension peak torque.

^f^ETP: extension total power.

#### Balance, Cardiopulmonary Endurance, and the IADL

Postintervention, both groups exhibited significant improvements in BBS and IADL scores (*P*<.05), while the improvements in TUGT and 6MWT scores were not statistically significant (Table 4). There were no significant differences between the groups in the changes observed in balance, cardiopulmonary endurance, or the IADL (Figure 4).

**Table 4:** Baseline, 4-week, and pre- and postintervention changes in balance, cardiopulmonary endurance, and the IADL^a^.

Balance indicators	TRG^b^ (n=24)					IRG^c^ (n=27)					Between-group difference *P* value
	Baseline, mean (SD)	Week 4, mean (SD)	Difference, mean (SD)	*P* value	Baseline, mean (SD)		Week 4, mean (SD)	Difference, mean (SD)	*P* value		
									
BBS^d^	49.00 (4.18)	52.19 (3.10)	3.19 (2.86)	<.001	49.11 (4.89)		52.15 (3.79)	3.06 (2.44)	<.001	.88	
TUGT^e^ (seconds)	9.23 (2.19)	8.57 (1.71)	-0.66 (1.44)	.09	9.68 (2.76)		8.94 (2.14)	-0.73 (1.79)	.10	.90	
6MWT^f^ (minutes)	354.19 (97.56)	363.19 (82.29)	8.28 (41.92)	.37	349.84 (97.43)		364.63 (112.27)	13.58 (40.72)	.11	.72	
IADL	21.31 (1.96)	21.81 (1.76)	0.50 (0.89)	.04	21.42 (1.98)		22.05 (1.58)	0.63 (1.06)	.02	.70	

^a^IADL: Instrumental Activities of Daily Living.

^b^TRG: telerehabilitation group.

^c^IRG: in-person rehabilitation group.

^d^BBS: Berg Balance Scale.

^e^TUGT: Timed Up-and-Go Test.

^f^6MWT: 6-Minute Walk Test.

## Discussion

### Principal Findings

With the increase in life expectancy and the decline in birth rates worldwide, the proportion of older adults in China has risen sharply over the past few decades. As the aging population grows, national health care expenditures have also surged, prompting the Chinese government to prioritize addressing the health challenges associated with population aging. Sarcopenia, characterized by a significant decline in muscle mass due to factors such as nutritional status, physical activity, genetics, or hormonal changes, along with the deterioration of tendon performance and neural patterns [22], leads to a loss of muscle strength and mobility. Sarcopenia is one of the most critical factors contributing to functional decline and loss of independence in older adults. It not only reduces their quality of life but is also associated with increased morbidity and mortality, as well as elevated public health costs [22]. Supervised exercise is considered an effective strategy for managing sarcopenia and frailty, yet many patients lack access to such resources. This study found that a 4-week remote resistance training program significantly improves the strength and balance of patients with sarcopenia, with outcomes comparable to face-to-face rehabilitation supervised by therapists. These findings highlight the potential of telerehabilitation as a feasible solution for populations with limited access to health care resources and demonstrate how digital health solutions can enhance care for older adults and address the unique needs of an aging society.

Previous studies have reported mixed findings on the effects of resistance training on body composition. Some scholars have observed that 12 weeks of resistance training can increase muscle mass in patients with sarcopenic [23,24]. In another study, women with obesity-related sarcopenia underwent 12 weeks of resistance training, 3 times per week. Although the training group experienced an increase in the lean mass of the lower limbs, the increase in the total body lean mass did not reach statistical significance compared to the control group [25]. Another study found that after 3 months of resistance training in older adults with obesity-related sarcopenia, reductions were observed in the fat mass, total fat mass, and BFP in the upper limbs, but there was no significant increase in lean mass [26]. Two systematic reviews and meta-analyses also yielded conflicting conclusions [9,10]. Mechanistically, the increase in muscle mass in older adults following resistance training may be attributed to enhanced muscle protein synthesis [27], increased satellite cell activity and quantity [28], elevated secretion of anabolic hormones [29], improved mitochondrial quality and function [30], and decreased activity of catabolic cytokines [31]. In our study, we found both groups demonstrated an increase in lean mass and a decrease in the BFP, though these changes did not reach statistical significance. This may be due to the relatively short duration of the 4-week resistance training intervention, which may not have been sufficient to induce significant alterations in body composition. It is also possible that extending the training period could result in more pronounced changes in body composition.

In this study, both the TRG and the IRG showed significant improvements in strength-related indicators, including grip strength, 30SSRT and 30SACT scores, and knee extension torque indices. These findings are consistent with previous systematic reviews and meta-analyses [9,10]. Studies have shown that resistance training can increase muscle fiber volume, particularly type II fast-twitch fibers [32]. Similarly, Ruiz et al [33] and Nunes et al [34] confirmed that resistance training enhances muscle function by improving muscle fiber adaptability and optimizing strength output mechanisms. At the molecular level, Sousa-Victor et al [35] demonstrated that resistance training increases satellite cell activity and muscle protein synthesis, reduces muscle degradation, and improves muscle mass. Coletti et al [36] and Gadelha et al [37] showed that this type of exercise activates the mammalian target of rapamycin (mTOR) signaling pathway, influencing protein synthesis, autophagy, and the expression of peroxisome proliferator–activated receptor-gamma coactivator 1 alpha (PGC-1α), thereby promoting sustained muscle growth and enhancing muscle performance. In this study, these effects were observed after just 4 weeks of resistance training, further demonstrating the significant therapeutic role of resistance training in the treatment of sarcopenia. The lack of significant differences in training outcomes between the 2 groups supports the application of telerehabilitation therapies in older adults with sarcopenia.

Lower limb muscle endurance is critical for dynamic balance. The reduction in the muscle cross-sectional area and neural fibers, particularly fast-twitch fibers, leads to a decline in muscle strength, especially in the lower limbs. This decrease in lower limb strength causes patients to spend extended periods sitting or lying down. Prolonged inactivity and sedentary behavior further exacerbate muscle loss and strength decline [25,35]. Reduced lower limb function impairs the ability to perform daily tasks, such as standing up from a chair, picking up items from the floor, walking, and climbing stairs [38]. In situations of instability, the central nervous system compensates by increasing muscle recruitment. One study [39] found that with aging, there is a loss of up to 75% of type II fibers, which diminishes functional mobility and muscle strength output. Concurrently, the synaptic input to α-motor neurons and the number of cortical neurons forming the corticospinal tract also decrease [40]. Resistance training can activate more motor units and muscle fibers, enhance synaptic input to α-motor neurons, and recruit more cortical neurons within the corticospinal tract, thereby improving coordination, reaction time, and strength to support dynamic balance [41,42]. Studies [43,44] have demonstrated that 3 months of resistance training improves muscle mass, physical fitness, and functional outcomes. In another study [11], participants’ functional fitness, such as the walking time, the chair stand test score, and the 8-foot up-and-go score, improved compared to the control group. In this study, after 4 weeks of training, both groups showed improvements in their BBS scores, which is consistent with previous findings. However, there was no significant improvement in TUGT scores, likely due to the relatively short duration of the training. The effective stimulation of key muscle groups, such as the gluteus maximus, quadriceps, and tibialis anterior, by resistance training may improve lower limb strength and neuromuscular coordination, enhancing stability and balance.

In terms of cardiorespiratory endurance, although resistance training primarily focuses on muscle strengthening rather than endurance, research suggests that resistance training may also improve endurance. For patients with sarcopenia, increased lower limb strength can translate into faster walking speed [25], and training peripheral muscles may improve indices related to respiratory function, thereby providing more substantial support for endurance [45]. One study [46] indicated that resistance training could enhance the 6-minute walking distance in frail older adults, while another systematic review [47] found that resistance training improves cardiorespiratory function in healthy older adults, including the peak oxygen uptake, anaerobic threshold, and 6-minute walking distance. In this study, both groups demonstrated improvements in the 6-minute walking distance, though neither reached statistical significance, possibly due to the short duration of the training program. Regarding the IADL, previous systematic reviews have shown that patients with reduced muscle mass are more likely to experience impairments in activities of daily living (ADL) and IADL, with reduced grip strength also being associated with impairments in ADL and IADL [48]. In this study, both groups showed significant improvements in the IADL after training, with no significant differences observed between the groups.

### Limitations

One of the limitations of this study is that it was a single-center RCT with a relatively small sample size. To address this limitation, the research team plans to conduct follow-up multicenter studies in regions with limited health care resources. Additionally, the follow-up period was relatively short. Future studies will involve a larger cohort over 8 weeks, 12 weeks, or even longer. Lastly, although participants were instructed to maintain consistent dietary habits throughout the study, detailed nutritional data were not adequately recorded. In addition, participants were not blinded, which may have introduced bias. In this study, the TRG received one-time offline instruction from a physiotherapist and were provided with resistance bands of appropriate tension. Furthermore, the pressure of having a follow-up assessment 1 month later may have increased patient compliance compared to routine application settings.

### Conclusion

A 4-week mobile app–based telerehabilitation program produced improvements in muscle strength, balance, and the IADL comparable to those achieved by traditional in-person therapy in older adults with sarcopenia. Additionally, this telerehabilitation approach reduced health care resource use and logistical barriers. By offering an accessible, cost-effective, and patient-centered model of care, telerehabilitation shows promise as a viable alternative for patients with sarcopenia lacking convenient access to conventional rehabilitation services.

## Appendix

Multimedia Appendix 1 CONSORT (Consolidated Standards of Reporting Trials) checklist.

Multimedia Appendix 2 Remote home-based exercise protocol for patients with sarcopenia.

## Abbreviations

30SACT: 30-Second Arm Curl Test
30SSRT: 30-Second Sitting-to-Rising Test
6MWT: 6-Minute Walk Test
ADL: activities of daily living
BBS: Berg Balance Scale
BFP: body fat percentage
BIA: bioelectrical impedance analysis
EPT: extension peak torque
ETP: extension total power
IADL: Instrumental Activities of Daily Living
IRG: in-person rehabilitation group
RCT: randomized controlled trial
RPE: rating of perceived exertion
SMI: skeletal muscle mass index
TRG: telerehabilitation group
TSM: total skeletal muscle mass
TUGT: Timed Up-and-Go Test

## Data Availability

All data generated and analyzed during this study are fully presented in this paper and the multimedia appendices.

## References

[1] Addante F, Gaetani F, Patrono L, Sancarlo D, Sergi I, Vergari G (2019). An innovative AAL system based on IoT technologies for patients with sarcopenia. Sensors (Basel).

[2] Alhussain MH, Alkahtani S, Aljuhani O, Habib SS (2020). Effects of nutrient intake on diagnostic measures of sarcopenia among Arab men: a cross-sectional study. Nutrients.

[3] Wei M, Meng D, Guo H, He S, Tian Z, Wang Z, Yang G, Wang Z (2022). Hybrid exercise program for sarcopenia in older adults: the effectiveness of explainable artificial intelligence-based clinical assistance in assessing skeletal muscle area. Int J Environ Res Public Health.

[4] Buccheri E, Dell'Aquila D, Russo M, Chiaramonte R, Musumeci G, Vecchio M (2023). Can artificial intelligence simplify the screening of muscle mass loss?. Heliyon.

[5] Petermann-Rocha F, Balntzi V, Gray SR, Lara J, Ho FK, Pell JP, Celis-Morales C (2022). Global prevalence of sarcopenia and severe sarcopenia: a systematic review and meta-analysis. J Cachexia Sarcopenia Muscle.

[6] Chen L, Woo J, Assantachai P, Auyeung T, Chou M, Iijima K, Jang HC, Kang L, Kim M, Kim S, Kojima T, Kuzuya M, Lee JSW, Lee SY, Lee W, Lee Y, Liang C, Lim J, Lim WS, Peng L, Sugimoto K, Tanaka T, Won CW, Yamada M, Zhang T, Akishita M, Arai H (2020). Asian Working Group for Sarcopenia: 2019 consensus update on sarcopenia diagnosis and treatment. J Am Med Dir Assoc.

[7] Meng S, He X, Fu X, Zhang X, Tong M, Li W, Zhang W, Shi X, Liu K (2024). The prevalence of sarcopenia and risk factors in the older adult in China: a systematic review and meta-analysis. Front Public Health.

[8] Mcleod JC, Currier BS, Lowisz CV, Phillips SM (2024). The influence of resistance exercise training prescription variables on skeletal muscle mass, strength, and physical function in healthy adults: an umbrella review. J Sport Health Sci.

[9] Chen N, He X, Feng Y, Ainsworth BE, Liu Y (2021). Effects of resistance training in healthy older people with sarcopenia: a systematic review and meta-analysis of randomized controlled trials. Eur Rev Aging Phys Act.

[10] Talar K, Hernández-Belmonte Alejandro, Vetrovsky T, Steffl M, Kałamacka E, Courel-Ibáñez Javier (2021). Benefits of resistance training in early and late stages of frailty and sarcopenia: a systematic review and meta-analysis of randomized controlled studies. J Clin Med.

[11] Rodrigues F, Domingos C, Monteiro D, Morouço P (2022). A review on aging, sarcopenia, falls, and resistance training in community-dwelling older adults. Int J Environ Res Public Health.

[12] Sharma N, Chahal A, Balasubramanian K, Sanjeevi RR, Rai RH, Bansal N, Muthukrishnan R, Sharma A (2023). Effects of resistance training on muscular strength, endurance, body composition and functional performance among sarcopenic patients: a systematic review. J Diabetes Metab Disord.

[13] Fragala M, Cadore E, Dorgo S, Izquierdo M, Kraemer W, Peterson M (2019). Resistance training for older adults: position statement from the National Strength and Conditioning Association. J Strength Cond Res.

[14] Hurst C, Robinson S, Witham M, Dodds R, Granic A, Buckland C, De Biase S, Finnegan S, Rochester L, Skelton DA, Sayer AA (2022). Resistance exercise as a treatment for sarcopenia: prescription and delivery. Age Ageing.

[15] Vikberg S, Björk S, Nordström A, Nordström P, Hult A (2022). Feasibility of an online delivered, home-based resistance training program for older adults - a mixed methods approach. Front Psychol.

[16] (2020). Technical Advisory Group for measurement, monitoring and evaluation of the UN Decade of Healthy Ageing. World Health Organization.

[17] Haleem A, Javaid M, Singh RP, Suman R (2021). Telemedicine for healthcare: capabilities, features, barriers, and applications. Sens Int.

[18] Cavill NA, Foster CE (2018). Enablers and barriers to older people’s participation in strength and balance activities: a review of reviews. JFSF.

[19] Walters K, Frost R, Kharicha K, Avgerinou C, Gardner B, Ricciardi F, Hunter R, Liljas A, Manthorpe J, Drennan V, Wood J, Goodman C, Jovicic A, Iliffe S (2017). Home-based health promotion for older people with mild frailty: the HomeHealth intervention development and feasibility RCT. Health Technol Assess.

[20] van der Kolk NM, de Vries NM, Kessels RPC, Joosten H, Zwinderman AH, Post B, Bloem BR (2019). Effectiveness of home-based and remotely supervised aerobic exercise in Parkinson's disease: a double-blind, randomised controlled trial. Lancet Neurol.

[21] Tsekoura M, Billis E, Tsepis E, Dimitriadis Z, Matzaroglou C, Tyllianakis M, Panagiotopoulos E, Gliatis J (2018). The effects of group and home-based exercise programs in elderly with sarcopenia: a randomized controlled trial. J Clin Med.

[22] Yuan S, Larsson SC (2023). Epidemiology of sarcopenia: prevalence, risk factors, and consequences. Metabolism.

[23] Hong J, Kim J, Kim SW, Kong H (2017). Effects of home-based tele-exercise on sarcopenia among community-dwelling elderly adults: body composition and functional fitness. Exp Gerontol.

[24] Bagheri R, Moghadam BH, Church DD, Tinsley GM, Eskandari M, Moghadam BH, Motevalli MS, Baker JS, Robergs RA, Wong A (2020). The effects of concurrent training order on body composition and serum concentrations of follistatin, myostatin and GDF11 in sarcopenic elderly men. Exp Gerontol.

[25] Liao C, Tsauo J, Lin L, Huang S, Ku J, Chou L (2017). Effects of elastic resistance exercise on body composition and physical capacity in older women with sarcopenic obesity: a CONSORT-compliant prospective randomized controlled trial. Medicine (Baltimore).

[26] Huang S, Ku J, Lin L, Liao C, Chou L, Liou T (2017). Body composition influenced by progressive elastic band resistance exercise of sarcopenic obesity elderly women: a pilot randomized controlled trial. Eur J Phys Rehabil Med.

[27] Schulte J, Yarasheski K (2001). Effects of resistance training on the rate of muscle protein synthesis in frail elderly people. Int J Sport Nutr Exerc Metab.

[28] Verdijk LB, Gleeson BG, Jonkers RAM, Meijer K, Savelberg HHCM, Dendale P, van Loon LJC (2009). Skeletal muscle hypertrophy following resistance training is accompanied by a fiber type-specific increase in satellite cell content in elderly men. J Gerontol A Biol Sci Med Sci.

[29] Smilios I, Pilianidis T, Karamouzis M, Parlavantzas A, Tokmakidis S (2007). Hormonal responses after a strength endurance resistance exercise protocol in young and elderly males. Int J Sports Med.

[30] Burtscher J, Soltany A, Visavadiya NP, Burtscher M, Millet GP, Khoramipour K, Khamoui AV (2023). Mitochondrial stress and mitokines in aging. Aging Cell.

[31] Cornish SM, Chilibeck PD (2009). Alpha-linolenic acid supplementation and resistance training in older adults. Appl Physiol Nutr Metab.

[32] Beurskens R, Gollhofer A, Muehlbauer T, Cardinale M, Granacher U (2015). Effects of heavy-resistance strength and balance training on unilateral and bilateral leg strength performance in old adults. PLoS One.

[33] Ruiz JR, Sui X, Lobelo F, Morrow JR, Jackson AW, Sjöström M, Blair SN (2008). Association between muscular strength and mortality in men: prospective cohort study. BMJ.

[34] Nunes EA, Stokes T, McKendry J, Currier BS, Phillips SM (2022). Disuse-induced skeletal muscle atrophy in disease and nondisease states in humans: mechanisms, prevention, and recovery strategies. Am J Physiol Cell Physiol.

[35] Sousa-Victor P, García-Prat L, Muñoz-Cánoves P (2022). Control of satellite cell function in muscle regeneration and its disruption in ageing. Nat Rev Mol Cell Biol.

[36] Coletti C, Acosta GF, Keslacy S, Coletti D (2022). Exercise-mediated reinnervation of skeletal muscle in elderly people: an update. Eur J Transl Myol.

[37] Gadelha AB, Paiva FML, Gauche R, de Oliveira RJ, Lima RM (2016). Effects of resistance training on sarcopenic obesity index in older women: a randomized controlled trial. Arch Gerontol Geriatr.

[38] Philp A, Hamilton DL, Baar K (2011). Signals mediating skeletal muscle remodeling by resistance exercise: PI3-kinase independent activation of mTORC1. J Appl Physiol (1985).

[39] Keller K, Engelhardt M (2019). Strength and muscle mass loss with aging process. Age and strength loss. Muscle Ligaments Tendons J.

[40] Bruyere O, Wuidart M, Di Palma E, Gourlay M, Ethgen O, Richy F, Reginster J (2005). Controlled whole body vibration to decrease fall risk and improve health-related quality of life of nursing home residents. Arch Phys Med Rehabil.

[41] Liao C, Tsauo J, Huang S, Ku J, Hsiao D, Liou T (2018). Effects of elastic band exercise on lean mass and physical capacity in older women with sarcopenic obesity: a randomized controlled trial. Sci Rep.

[42] Hruda KV, Hicks AL, McCartney N (2003). Training for muscle power in older adults: effects on functional abilities. Can J Appl Physiol.

[43] Boiko Ferreira LH, Schoenfeld BJ, Smolarek AC, McAnulty SR, Mascarenhas LPG, Souza Junior TP (2021). Effect of 12 weeks of resistance training on motor coordination and dynamic balance of older woman. Rejuvenation Res.

[44] Tuan S, Chang L, Sun S, Li C-H, Chen G, Tsai Y (2024). Assessing the clinical effectiveness of an exergame-based exercise training program using Ring Fit Adventure to prevent and postpone frailty and sarcopenia among older adults in rural long-term care facilities: randomized controlled trial. J Med Internet Res.

[45] Flor-Rufino C, Barrachina-Igual J, Pérez-Ros P, Pablos-Monzó A, Martínez-Arnau FM (2023). Resistance training of peripheral muscles benefits respiratory parameters in older women with sarcopenia: randomized controlled trial. Arch Gerontol Geriatr.

[46] Lai X, Zhu H, Wu Z, Chen B, Jiang Q, Du H, Huo X (2023). Dose-response effects of resistance training on physical function in frail older Chinese adults: a randomized controlled trial. J Cachexia Sarcopenia Muscle.

[47] Smart TFF, Doleman B, Hatt J, Paul M, Toft S, Lund JN, Phillips BE (2022). The role of resistance exercise training for improving cardiorespiratory fitness in healthy older adults: a systematic review and meta-analysis. Age Ageing.

[48] Wang DX, Yao J, Zirek Y, Reijnierse EM, Maier AB (2020). Muscle mass, strength, and physical performance predicting activities of daily living: a meta-analysis. J Cachexia Sarcopenia Muscle.

