# Supplementary material for: A 4-Week Mobile App–Based Telerehabilitation Program vs Conventional In-Person Rehabilitation in Older Adults With Sarcopenia: Randomized Controlled Trial
Source: J Med Internet Res. 2025 Jan 24;27:e67846. doi: 10.2196/67846 (PMC11806269; doi:10.2196/67846)
Supplement: Multimedia Appendix 2 [file jmir_v27i1e67846_app2.docx]

Remote Home Exercise Principles

1, All exercises should be completed without any pain, dizziness, or nausea, etc. if a certain exercise causes discomfort or other symptoms, then this exercise should be given up.

2, All exercises need to be completed gently and slowly, avoiding any fast or violent motion!

3, Do not hold breathing during the maintenance phase with all exercises, so as to avoid a sudden increase in blood pressure. Counting out loudly is an efficient method to avoid holding breath.

4, All exercises must be measured, gradually increase the intensity in order to avoid sports injuries!

**The frequency for each exercise: three sets of 10, three times a week.**

| **GLUTE BRIDGE** | **GLUTE BRIDGE**  **Lie on the ground with your knees bent and your feet flat on the ground, hip-width apart. Push through your heels and lift your hips until your knees, hips, and shoulders form a straight line. Hold at the top for 1-2 seconds, then slowly lower back to the starting position** | **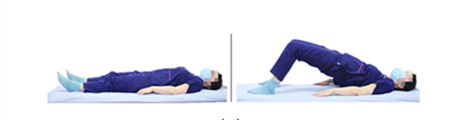** |
| --- | --- | --- |
| **RESISTED ELBOW FLEXION** | **RESISTED ELBOW FLEXION**  **First, you need to prepare a resistance band and stand up straight.**  **Then, place one end of the band under your foot and wrap the other end around your hand once, holding it with your palm facing up.**  **Next, bend your elbows with the maximum force while keeping your shoulders still, and hold this position for 6-8 seconds.**  **If you experience pain in your elbows, practice according to your own condition. This exercise can also be completed while sitting on a chair.** | **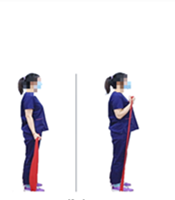** |
| **RESISTED HIP EXTENSION** | **RESISTED HIP EXTENSION**  **First, you need to stand in front of a table or wall with a sturdy handrail, hold onto the table or wall with both hands, and open your feet to shoulder-width.**  **Then, fasten the resistance band and loop it around your ankles.**  **Next, you need to extend one leg backward with the maximum force, keeping the knee straight, until you feel a tightness in the back of your hips, and hold this position for 6-8 seconds. Afterward, slowly and controlledly lower your foot back to the ground.**  **If you have pain in your hip joints, practice according to your own condition.** | **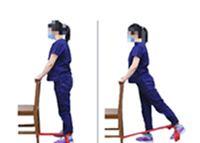** |
| **RESISTED HIP ABDUCTION** | **RESISTED HIP ABDUCTION**  **First, you need to stand in front of a table or wall with a sturdy handrail, hold onto the table or wall with both hands, and open your feet to shoulder-width.**  **Then, fasten the resistance band and loop it around your ankles.**  **Next, you will extend one leg outward to the side with maximum force, keeping the knee straight, until you feel a tightness on the outer side of your hip, and hold this position for 6-8 seconds. Afterward, slowly and controlledly lower your foot back to the ground.**  **If you have pain in your hip joints, practice according to your own condition.** | **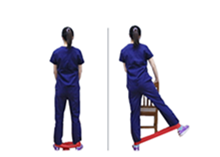** |
| **RESISTED SHOULDER ABDUCTION** | **RESISTED SHOULDER ABDUCTION**  **First, you need to prepare a resistance band and sit up straight on a chair with your feet flat on the ground.**  **Then, place one end of the band under your foot and wrap the other end around your hand once, holding it with your palm facing down.**  **Next, you need to lift your arms upward with the maximum force, keeping your elbows straight and your upper arms as close to your ears as possible, and hold this position for 6-8 seconds.Afterward, slowly and controlledly lower your upper arms back to their original position.**  **If you experience pain in your shoulders or arms, practice according to your own condition.** | 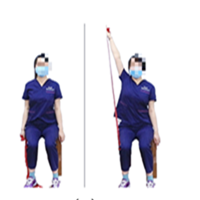 |
| **RESISTED KNEE EXTENSION** | **RESISTED KNEE EXTENSION**  **First, you need to prepare a resistance band and sit on a chair that is of moderate height and made of a firm material, sitting up straight with your legs naturally resting on the ground and your toes pointing forward.**  **Then, loop one end of the resistance band around your ankle and secure the other end to the leg of the chair on the same side.**  **Next, you need to extend your knee with maximum force and hold this position for 6-8 seconds. Afterward, slowly and controlledly bend your leg back to the starting position.**  **If you experience pain in your knee joints, practice according to your own condition.** | 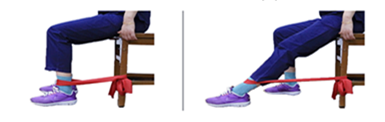 |
